# Prospective association of the Mediterranean diet with cardiovascular disease incidence and mortality and its population impact in a non-Mediterranean population: the EPIC-Norfolk study

**DOI:** 10.1186/s12916-016-0677-4

**Published:** 2016-09-29

**Authors:** Tammy Y. N. Tong, Nicholas J. Wareham, Kay-Tee Khaw, Fumiaki Imamura, Nita G. Forouhi

**Affiliations:** 1 MRC Epidemiology Unit, University of Cambridge School of Clinical Medicine, Cambridge, UK; 2 Department of Public Health and Primary Care, University of Cambridge, Cambridge, UK

## Abstract

**Background:**

Despite convincing evidence in the Mediterranean region, the cardiovascular benefit of the Mediterranean diet is not well established in non-Mediterranean countries and the optimal criteria for defining adherence are unclear. The population attributable fraction (PAF) of adherence to this diet is also unknown.

**Methods:**

In the UK-based EPIC-Norfolk prospective cohort, we evaluated habitual diets assessed at baseline (1993–1997) and during follow-up (1998–2000) using food-frequency questionnaires (n = 23,902). We estimated a Mediterranean diet score (MDS) using cut-points projected from the Mediterranean dietary pyramid, and also three other pre-existing MDSs. Using multivariable-adjusted Cox regression with repeated measures of MDS and covariates, we examined prospective associations between each MDS with incident cardiovascular diseases (CVD) by 2009 and mortality by 2013, and estimated PAF for each outcome attributable to low MDS.

**Results:**

We observed 7606 incident CVD events (2818/100,000 person-years) and 1714 CVD deaths (448/100,000). The MDS based on the Mediterranean dietary pyramid was significantly associated with lower incidence of the cardiovascular outcomes, with hazard ratios (95 % confidence intervals) of 0.95 (0.92–0.97) per one standard deviation for incident CVD and 0.91 (0.87–0.96) for CVD mortality. Associations were similar for composite incident ischaemic heart disease and all-cause mortality. Other pre-existing MDSs showed similar, but more modest associations. PAF due to low dietary pyramid based MDS (<95th percentile) was 3.9 % (1.3–6.5 %) for total incident CVD and 12.5 % (4.5–20.6 %) for CVD mortality.

**Conclusions:**

Greater adherence to the Mediterranean diet was associated with lower CVD incidence and mortality in the UK. This diet has an important population health impact for the prevention of CVD.

**Electronic supplementary material:**

The online version of this article (doi:10.1186/s12916-016-0677-4) contains supplementary material, which is available to authorized users.

## Background

The Mediterranean diet describes the traditional diet of Mediterranean regions such as Crete, other parts of Greece and Southern Italy [1–3]. The diet is typically high in the consumption of cereals, fruits, vegetables, legumes and olive oil, low in red meats, and moderate in the consumption of fermented dairy products, fish, poultry and wine [2, 4]. Since its recognition, adherence to the Mediterranean diet has been reported to be associated with lower incidence of non-communicable diseases, including cardiovascular diseases (CVD), cancer, neurodegenerative diseases and mortality [5–9]. Results from two randomised controlled trials also demonstrated the causal protective role of the diet in high-risk populations [8–11].

Although studies on the potential cardiovascular benefits of the Mediterranean diet have been published in both Mediterranean and non-Mediterranean cohorts, evidence from non-Mediterranean regions is less consistent. A Swedish cohort, for example, observed that high adherence to the Mediterranean diet was associated with lower cardiovascular mortality only among women [12], while in an Australian cohort, the association was observed only among men [13]. An Eastern European study also showed that high adherence to a Mediterranean diet was associated with lower all cause and CVD mortality, but not with ischaemic heart disease (IHD) or stroke mortality [14]. In the United Kingdom (UK), although the UK National Institute for Health and Care Excellence recommends a Mediterranean style diet for the secondary prevention of CVD, no study in the country has examined the association of adherence to the Mediterranean diet with incident CVD [15].

Moreover, in assessing adherence to the Mediterranean diet, published studies evaluated different Mediterranean diet scores (MDSs) [16–21], but there is sparse evidence on whether or not each algorithm would be useful in non-Mediterranean countries. In addition, most published MDSs did not take into account the current recommendations for adopting the Mediterranean diet [22]. Finally, the population impact of a cardiovascular benefit from adhering to the Mediterranean diet at the general population level also remains unknown, partly because the previous trials may have limited generalisability by recruiting highly selected adults only.

Therefore, we aimed to assess the association of the Mediterranean diet as defined by different MDSs with incident CVD, CVD mortality and all-cause mortality in a UK-based cohort, and to estimate the population attributable fraction (PAF) for cardiovascular and mortality outcomes for low adherence to the Mediterranean diet.

## Methods

### Study population and design

European Prospective Investigation of Cancer (EPIC)-Norfolk is an ongoing UK-based prospective cohort and part of the Europe-wide multi-centre EPIC study. Details of the study design were described previously [23]. Briefly, 25,639 men and women aged 40–79 in eastern England were recruited through general practice registers and underwent baseline assessment between 1993 and 1997. Participants were further invited to the follow-up assessment (1998 to 2000), and were followed up by 2009 for incident outcomes and by 2013 for mortality. At the baseline and follow-up visits, the participants were asked to complete a health and lifestyle questionnaire and a food frequency questionnaire (FFQ). We excluded 644 participants who did not complete any FFQs. In the analysis for incident CVD, we additionally excluded 1093 participants who reported myocardial infarction (n = 790) or stroke (n = 303) at baseline. This study thus evaluated 23,902 participants (n = 11,258 with dietary data at both baseline and follow-up, and n = 208 with dietary data at follow-up only). Ethical approval for the study was obtained from the Norwich District Ethics Committee and participants gave informed consent.

### Dietary assessment and Mediterranean diet scores (MDSs)

Habitual diet was assessed using a 130-item semi-quantitative FFQ which asked about participants’ average intake of the food items over the past year. Validity of this FFQ for major foods and nutrients was previously assessed against 16-day weighted dietary records, 24-hour recall and selected biomarkers in the sub-sample of EPIC-Norfolk [24–26]. Reproducibility of the assessment of dietary components typical of the Mediterranean diet and the MDSs were evaluated in this study. For MDS calculation, we evaluated dietary intakes adjusted to a 2000 kcal/day (8.37 MJ/day) diet using the residual method to assess diet quality independent of diet quantity, and to partly reduce measurement errors because energy intake is partly related to under- or over-reporting of dietary consumption [27].

In this study, we evaluated four MDSs as measures of adherence to the Mediterranean diet. The algorithm for each is summarised in Additional file 1: Text S1 and Table S1. As the primary exposure, we evaluated a MDS based on the Mediterranean diet pyramid (pyramid-based MDS, PyrMDS). The pyramid was recently proposed by the Mediterranean Diet Foundation [4] to be applied to both Mediterranean and non-Mediterranean regions, with dietary guidelines accounting for the traditional Mediterranean diet and also the contemporary food environment [4]. We newly developed the algorithm to calculate the PyrMDS (Additional file 1: Table S2). The other three MDSs were identified through our systematic search for quantitative review articles published by January 12, 2016, on the Mediterranean diet and non-communicable diseases. We identified 254 unique records and retrieved 31 full-text articles. An algorithm for one MDS was recently developed by Sofi et al. [5] from their review of published literature on the Mediterranean diet (literature-based MDS, LitMDS). Whereas PyrMDS and LitMDS account for absolute levels of dietary consumption, the other two MDSs [17, 28], the most commonly used MDSs in the literature, assigned component scores based on cohort medians (mMDS) or tertiles of dietary consumption (tMDS) (Additional file 1: Text S1 and Table S1).

### Outcome ascertainment

The primary outcome was incident CVD, which included any first ever case of both non-fatal or fatal events due to IHD, ischaemic stroke, haemorrhagic stroke, heart failure, peripheral vascular disease, or other cardiovascular outcomes described by the relevant ICD codes (ICD9 401–448 or ICD10 I10–I79) [29]. Cause-specific hospital admission was determined using East Norfolk Commission Record of the National Health Service [29, 30]. Incident CVD was ascertained until March 31, 2009. CVD mortality and all-cause mortality, treated as a secondary outcome in this study, was confirmed via death certificates with ICD codes held at the UK Office for National Statistics and ascertained until June 30, 2013 [29, 30].

### Assessment of other covariates

Demographic, lifestyle and health characteristics were assessed at baseline and follow-up using a self-administered questionnaire. Physical activity levels were self-reported and characterised as a validated 4-point index [31]. Trained nurses measured each participant’s weight, height, waist circumference and blood pressure (systolic and diastolic) at each visit, and took non-fasting bloods from which blood lipids were assayed.

### Statistical analyses

Linear regression was used for descriptive analyses of associations between the MDSs and cohort baseline characteristics. Spearman’s rank correlation coefficients were assessed to examine reproducibility over time for MDSs and relevant dietary factors. In longitudinal analysis, repeated measures of diet and covariates were used, wherever possible, through the cumulative-average method [27]. We modelled Cox proportional-hazards regression to estimate hazard ratio (HR) and 95 % confidence interval (CI) for each of the selected outcomes. The underlying time variable was age from the first available FFQ to age at diagnosis of CVD (or death for mortality outcomes), or the date of administrative censoring, whichever occurred first. Each of the four MDSs was modelled continuously per standard deviation (SD) and categorically (three groups: low, medium or high adherence) with approximately equal numbers of participants in each group. Analyses were adjusted for potential confounders, and additionally for potential physiological mediators. Variables considered as potential confounders were age, sex, education level, social class, marital status, smoking, physical activity, season of FFQ assessment, body mass index (BMI), waist circumference, prevalent diabetes, medication use (anti-hypertensive drugs, lipid-lowering drugs, and hormone replacement therapy for women), and family history of diseases (diabetes, myocardial infarction and stroke). Potential mediators included total cholesterol, high-density lipoprotein cholesterol, low-density lipoprotein cholesterol, log triglycerides, and systolic and diastolic blood pressure. The proportional hazard assumption for MDSs was not rejected on the basis of Schoenfeld residuals in multivariable-adjusted Cox model. We performed additional analyses modelling two MDSs simultaneously to test if one of the MDSs was more strongly associated with CVD than the other. Missing covariates were observed in ≤ 3.9 % of participants for socioeconomic and lifestyle variables (3.9 % for social class and < 1.4 % for the others), and in ≤ 9.6 % of participants for physiological markers (9.6 % for blood lipids, 0.2 % for blood pressure or anthropometry). The missing information was imputed simultaneously by conducting regression-based multiple imputation (n = 10). In all analyses unless specified, estimates from 10 datasets were pooled under Rubin’s rules [32].

We estimated PAFs for each outcome attributable to low Mediterranean diet adherence, for which we assumed the observed associations were causal. Results based on PyrMDS are presented in this report accounting for its strongest public-health importance based on the longitudinal analyses. We calculated PAF based on the formula of rate difference [33]: PAF = (*I*_0_ − *I*_i_)/*I*_0_, equivalent to *I*_0_ − *HR* × *dMDS*/*I*_0,_ where HR was estimated continuously with adjustment for potential confounders as aforementioned, *I*_0_ is observed incidence per 10,000 person-years, and *I*_i_ represents a hypothetical, ideal incidence if the population achieved high MDS (95th percentile) (*dMDS* = MDS_*ideal*_ – MDS_*observed*_). The CI of the PAF was derived from bootstrapping [34] to estimate HR and PAF iteratively (n resampling = 100, after confirming no difference in results between n = 100 and 1000). In addition, we repeated estimation of PAFs in a high-risk population only. A high risk was defined as having a 10-year CVD risk of 10 % or higher based on QRISK2 [35], by which a clinical intervention is recommended in the UK [36].

In analysis of any dietary scores, an observed association with a health outcome can be driven by one component of the score. To rule out this possibility and assess importance of combining multiple dietary components, we repeated the primary analysis for each MDS after sequentially excluding each Mediterranean diet component from the total score. Other sensitivity analyses include using FFQ assessed at baseline only without use of repeated measures of diet; including only participants who completed both FFQs and using repeated measures only; excluding participants with potential implausible energy intakes (extreme 1st or 5th percentile); and adjusting for censoring due to competing risks of non-CVD mortality [37]. We also repeated analyses by re-constructing MDSs by grouping food items differently to consider variations of the Mediterranean diet [2, 4], for example by including only wine in the alcohol component. To assess whether the association of MDSs with outcomes was independent of baseline risk of CVDs, we also additionally adjusted for QRISK2 as a covariate. All analyses were performed using Stata version 13.1 (Stata Corp, Texas, United States) and *P* values of < 0.05 were considered significant.

## Results

### Cohort characteristics

Associations of adherence to the Mediterranean diet as defined by the four MDSs with baseline characteristics (sociodemographic, anthropometric, health and lifestyle) were similar across the scores (Table 1). Participants with high adherence were less likely to be current smokers, and more likely to be physically active and have a college education and higher social status compared to participants with low adherence. The primary MDS, PyrMDS, was moderately reproducible over 3.7 years (Spearman’s *ρ* = 0.60) (Additional file 1: Table S3) and correlated with the other MDSs (*ρ* = 0.53 with mMDS to *ρ* = 0.81 with tMDS). The Mediterranean diet components showed moderate reproducibility (*ρ* = 0.47 to 0.85 over).

**Table 1:** Cohort characteristics according to adherence to the Mediterranean diet at baseline of the EPIC-Norfolk Study (n = 23,902)

Characteristics	Mediterranean diet score (MDS), basis of scoring^a^							
	The Mediterranean diet pyramid (PyrMDS)		Published literature (LitMDS)		Medians of dietary intake (mMDS)		Tertiles of dietary intake (tMDS)	
	Low	High	Low	High	Low	High	Low	High
	n = 7898	n = 7898	n = 7730	n = 8351	n = 7903	n = 6266	n = 8927	n = 8574
Age (years)	59.4 (9.4)	58.2 (9.0)^c^	58.8 (9.4)	58.6 (9.1)	59.2 (9.3)	58.4 (9.1)^c^	59.1 (9.3)	58.7 (9.1)^c^
Sex, men (%)	56	32^c^	58	32^c^	44	45	46	42^c^
Education level (%)								
School until age 16 years	10	11	10	10	11	11	10	10
School until age 18 years	38	42	40	42	38	43	38	43
Bachelor’s degree or above	8	19^c^	10	16^c^	10	17^c^	10	17^c^
Marital status, married (%)	84	79^c^	83	80^c^	80	83	81	82
Smoking status (%)								
Current	16	8	17	8	16	7	15	8
Former	42	41^c^	42	41^c^	38	45^c^	38	45^c^
Physical activity level (%)								
Moderately inactive	26	31	27	30	28	29	28	30
Moderately active	23	24	23	24	22	24	22	24
Active	19	19^c^	19	19^c^	17	22^c^	17	20^c^
Occupational status (%)								
Unskilled worker	6	3	5	4	6	3	6	3
Skilled worker	63	52	60	54	61	53	61	51
Manager or equivalent	28	39	30	38	29	38	28	39
Professional	4	5^c^	4	5^c^	4	5^c^	4	5^c^
Family history of diabetes (%)	13	13	13	13	13	14	13	13
Family history of MI (%)	34	39^c^	34	38^c^	34	39^c^	35	38^c^
Family history of stroke (%)	24	25	24	25	24	24	24	24
Season of FFQ administered (%)								
Spring	27	27	26	28	26	27	27	27
Summer	24	26	24	26	25	26	25	25
Autumn	25	25^b^	26	25	25	25	25	25
Prevalent diabetes (%)	3	3	2	4^c^	3	3^b^	2	3^c^
Use of anti-hypertensive drug (%)	16	16	15	17^c^	16	17	16	17^b^
Use of lipid-lowering drug (%)	1	1^c^	1	2^c^	1	2^c^	1	2^c^
Use of HRT among women (%)	31	43^c^	29	43^c^	38	35^c^	37	37^c^
Body mass index, kg/m^2^	26.4 (3.8)	26.1 (3.9)	26.3 (3.7)	26.2 (4.0)	26.3 (4.0)	26.3 (3.8)	26.2 (3.9)	26.2 (3.9)
Waist circumference, cm	90 (12)	86 (12)^c^	90 (12)	86 (12)^c^	88 (12)	88 (12)^c^	88 (12)	87 (13)^c^
Systolic BP, mmHg	137 (18)	133 (18)^c^	136 (18)	134 (19)^c^	136 (18)	135 (18)^c^	136 (18)	135 (19)^c^
Diastolic BP, mmHg	83 (11)	81 (11)^c^	83 (11)	82 (11)^c^	83 (11)	82 (11)^c^	83 (11)	82 (11)^c^
Total cholesterol, mmol/L	6.2 (1.2)	6.2 (1.2)	6.2 (1.2)	6.2 (1.2)	6.2 (1.2)	6.1 (1.1)^c^	6.2 (1.2)	6.2 (1.2)
HDL cholesterol, mmol/L	1.4 (0.4)	1.5 (0.5)^c^	1.4 (0.4)	1.5 (0.4)^c^	1.4 (0.4)	1.5 (0.5)^c^	1.4 (0.4)	1.5 (0.5)^c^
LDL cholesterol, mmol/L	4.0 (1.0)	3.9 (1.1)^c^	4.0 (1.0)	3.9 (1.1)	4.0 (1.0)	3.9 (1.0)^c^	4.0 (1.0)	3.9 (1.0)^b^
Triglycerides, mmol/L	1.9 (1.2)	1.7 (1.0)^c^	1.9 (1.1)	1.7 (1.0)^c^	1.8 (1.1)	1.8 (1.1)^c^	1.9 (1.1)	1.7 (1.1)^c^
QRISK2 score, %^d^	19.4 (13.3)	16.5 (12.4)^c^	18.7 (13.1)	17.3 (12.7)^c^	18.8 (13.3)	17.3 (12.7)^c^	18.6 (13.1)	17.6 (12.9)^c^

EPIC, European Prospective Investigation of Cancer; FFQ, food frequency questionnaire; BP, blood pressure; HDL, high-density lipoprotein; LDL, low-density lipoprotein; HRT, hormone replacement therapy; MI, myocardial infarction

^a^Summary statistics in mean (SD) or percentages for extreme groups of thirds (assigned ensuring approximately equal numbers of observations per group) of each Mediterranean diet scores based on 23,694 participants with baseline FFQ. For each of the Mediterranean diet scores, ordinal scores were assigned to participants (see methods, Additional file 1: Table S1 and S2 for details)

^b^ *P* < 0.05 and ^c^ *P* < 0.01 for an association between a Mediterranean diet score and each row variable

^d^Predicts 10 year risk (%) of cardiovascular diseases

### Association of adherence to the Mediterranean diet with incident CVD and mortality

Of 23,902 participants, 7606 developed primary incident CVD (non-fatal or fatal) over 269,935 person-years (12.2 years of follow-up on average). A total of 5660 participants died over 382,765 person-years (17.0 years of follow-up on average), among whom 1714 deaths were due to CVD. Greater adherence to the Mediterranean diet was significantly associated with lower incidence of CVD in different multivariable-adjusted models (Table 2). For example, after adjustment for potential confounders, the HR per SD difference in PyrMDS was 0.95 (95 % CI, 0.93–0.97). The LitMDS and tMDS showed similar results, with an HR of 0.96 (95 % CI, 0.94–0.97) and 0.97 (95 % CI, 0.94–0.99), respectively, whereas mMDS was not significantly associated with incident CVD in any models (*P* trend > 0.05). Similar trends were observed when we additionally examined the HRs for quintiles of each MDS (Additional file 1: Table S4). In exploratory analyses comparing the four MDSs for prediction of CVD, PyrMDS, LitMDS and tMDS appeared similarly predictive of CVD, but superior to mMDS (Additional file 1: Table S5).

**Table 2:** Prospective association between adherence to the Mediterranean diet and incident cardiovascular diseases in EPIC-Norfolk (n = 23,902, 7606 cases, 269,935 person-years)

Mediterranean diet score^a^	Hazard ratios (95 % confidence intervals)		
	Adjusted for age and sex	Further adjusted for potential confounders^b^	Further adjusted for potential mediators^b^
PyrMDS, based on dietary pyramid (0–15)			
Low (3.2–8.0)	Reference	Reference	Reference
Medium (8.0–9.1)	0.95 (0.90–1.00)	0.96 (0.91–1.02)	0.97 (0.92–1.02)
High (9.1–13.1)	0.85 (0.80–0.90)	0.89 (0.84–0.94)	0.91 (0.85–0.96)
*P* trend	<0.001	<0.001	0.001
Per SD difference	0.93 (0.91–0.95)	0.95 (0.92–0.97)	0.95 (0.93–0.97)
LitMDS, based on literature (0–18)			
Low (0–8)	Reference	Reference	Reference
Medium (9–10)	0.96 (0.90–1.01)	0.95 (0.90–1.01)	0.95 (0.90–1.01)
High (11–18)	0.91 (0.86–0.97)	0.92 (0.87–0.97)	0.92 (0.87–0.98)
*P* trend	0.002	0.005	0.005
Per SD difference	0.96 (0.93–0.98)	0.96 (0.94–0.99)	0.96 (0.94–0.98)
mMDS, based on medians (0–9)			
Low (0–3)	Reference	Reference	Reference
Medium (4–5)	0.94 (0.89–0.99)	0.96 (0.91–1.01)	0.95 (0.90–1.00)
High (6–9)	0.95 (0.89–1.00)	0.97 (0.92–1.03)	0.97 (0.91–1.03)
*P* trend	0.053	0.295	0.21
Per SD difference	0.97 (0.95–1.00)	0.98 (0.96–1.01)	0.98 (0.96–1.00)
tMDS, based on tertiles (0–18)			
Low (0–7)	Reference	Reference	Reference
Medium (8–9)	0.97 (0.92–1.03)	0.98 (0.93–1.04)	0.97 (0.92–1.03)
High (10–18)	0.93 (0.88–0.98)	0.94 (0.89–0.99)	0.93 (0.88–0.98)
*P* trend	0.008	0.024	0.011
Per SD difference	0.96 (0.94–0.98)	0.97 (0.94–0.99)	0.96 (0.94–0.99)

^a^For each Mediterranean diet score, three groups (low, medium and high adherence) were assigned to ensure approximately equal numbers of observations per group. Ordinal scores were assigned to participants, according to four different pre-specified algorithms (see methods, Additional file 1: Table S1 and S2 for details)

^b^See methods for list of confounders and mediators

**Fig. 1:**
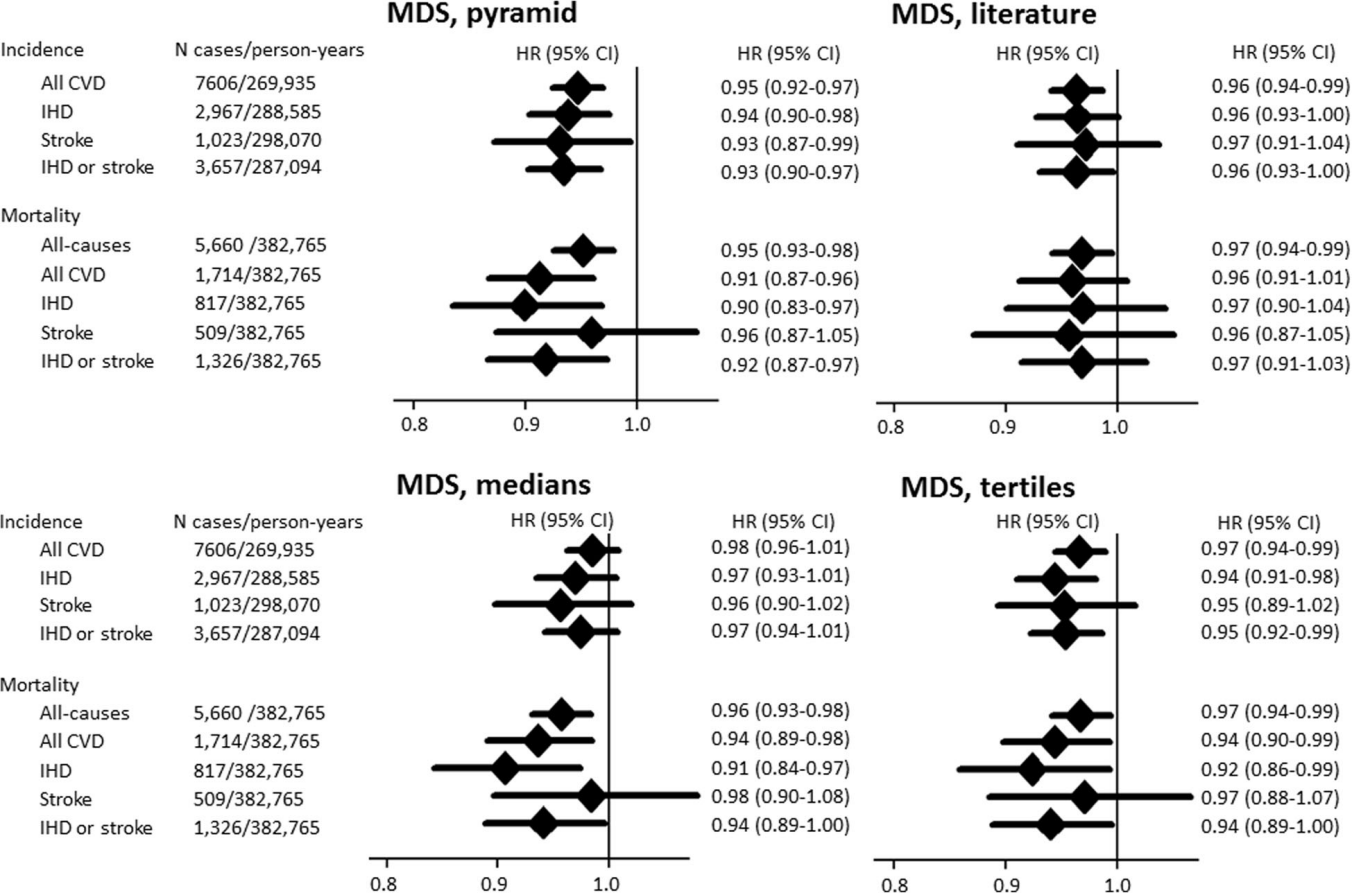
Prospective associations of Mediterranean diet adherence with cardiovascular diseases and all-cause mortality in EPIC-Norfolk (n = 23,902). CVD, cardiovascular diseases; IHD, ischaemic heart disease. Hazard ratio (HR) and 95 % confidence interval (CI) were estimated per one standard deviation of each of the four Mediterranean diet scores (MDSs). All estimates were estimated with adjustment for confounders

When we examined the association of adherence to the Mediterranean diet with subtypes of primary CVD events (i.e. IHD and stroke separately) and cause-specific and all-cause mortality (Fig. 1), the trends in magnitude of associations were similar to that for all incident CVD. Overall, PyrMDS was associated with lower hazard of most outcomes examined. Per SD difference in PyrMDS, we observed a lower incidence of all-cause mortality (HR adjusted for potential confounders 0.95; 95 % CI, 0.93–0.98), CVD mortality (0.91; 0.87–0.96), incident IHD (0.94; 0.90–0.98), incident stroke (0.93; 0.87–0.99), incident composite IHD or stroke (0.93; 0.90–0.97), IHD mortality (0.90; 0.83–0.97), and composite IHD or stroke mortality (0.92; 0.87–0.97), but no significant association for stroke mortality (0.96; 0.87–1.05).

### Population attributable fraction

In the EPIC-Norfolk population, 3.9 % (95 % CI, 1.3–6.5 %) of total CVD was estimated to be attributable to low Mediterranean diet adherence (PyrMDS lower than its 95th percentile, 10.7 of 15 points) (Table 3). This was equivalent to 9.7 cases of total CVD preventable per 1000 population over 10 years. If considering incident IHD or stroke events, the PAF estimate was 8.5 % (3.1, 13.9 %), equivalent to 10.2 IHD or stroke cases preventable per 1000 population. For CVD mortality and all-cause mortality, PAFs in the whole cohort were 12.5 % (4.5, 20.6 %) and 5.4 % (1.3, 9.5 %), equivalent to 5.5 and 7.5 cases per 1000 population over 10 years, respectively. Amongst a high-risk population (QRISK2 score ≥ 10 %) (n = 15767), the corresponding PAF (95 % CI) for total incident CVD was 3.9 % (1.1, 6.7 %), equivalent to 13.0 cases preventable per 1000 population over 10 years. Estimates for adherence to PyrMDS lower than the top third, or 67th percentile (scoring 9.1 points out of 15) among the EPIC-Norfolk population were of a comparable magnitude (Additional file 1: Table S6).

**Table 3:** Cardiovascular diseases and all-cause mortality, the number of cases and proportion potentially preventable by increasing adherence to the Mediterranean diet: the EPIC-Norfolk study^a^

	The whole cohort (n = 23,902)			High risk population (n = 15,767)^b^		
	Incidence^c^	Cases preventable^c^	PAF% (95 % CI)^c^	Incidence^c^	Cases preventable^c^	PAF% (95 % CI)^c^
Incident CVD events						
All incident CVD	248.6	9.7	3.9 (1.3–6.5)	334.4	13.0	3.9 (1.1–6.7)
Incident IHD	98.2	8.4	8.5 (1.9–15.2)	138.3	10.8	7.8 (1.3–14.3)
Incident stroke	33.8	3.7	10.8 (−1.5 to 23.1)	48.1	4.9	10.2 (−2.1 to 22.6)
Incident IHD or stroke	120.3	10.2	8.5 (3.1–13.9)	168.9	13.1	7.7 (2.3–13.2)
Mortality events						
All-cause mortality	138.4	7.5	5.4 (1.3–9.5)	191.3	10.9	5.7 (1.6–9.8)
CVD mortality	43.9	5.5	12.5 (4.5–20.6)	65.0	7.4	11.4 (3.3–19.6)
IHD mortality	21.1	3.5	16.6 (1.9–31.2)	31.3	4.8	15.4 (0.5–30.3)
Stroke mortality	13.2	0.7	5.3 (−12.0 to 22.7)	19.6	0.9	4.6 (−13.0 to 22.2)
IHD or stroke mortality	34.1	4.1	11.9 (1.75–22.0)	50.3	5.4	10.7 (0.48–20.9)

^a^Increasing adherence to the top 5 % (95th percentile, or 10.7 out of possible 15 points) of the Mediterranean dietary score based on the dietary pyramid (PyrMDS). See Additional file 1: Table S2 for further details on the scoring criteria for PyrMDS

^b^High risk defined as a QRISK2 score of 10 % or above for 10 year risk of CVD, for whom a pharmacological intervention (statin treatment) is advised in the United Kingdom

^c^Per 1000 population over 10 years. PAF, indicating proportion of cases attributable to the exposure of interest (low adherence to the Mediterranean diet)

CI, confidence interval; CVD, cardiovascular disease; IHD, ischaemic heart disease; PAF, population attributable fraction

### Sensitivity analyses

In analyses using MDSs in which each Mediterranean diet component was sequentially excluded, significant inverse associations remained reasonably stable regardless of the excluded component, especially when adherence was assessed using either PyrMDS or LitMDS (Additional file 1: Figure S1). Results were also similar in analyses using baseline FFQ only, using averages of the two FFQs from follow-up onwards only, excluding outliers of total energy intake, or controlling for competing risks due to non-CVD mortality (Additional file 1: Table S7). The association was also unchanged when we modified food groupings, excluding non-fermented dairy products, processed fish, refined cereal products, and alcohol other than wine, or when we additionally adjusted for QRISK2.

## Discussion

Our study is the first report on the association of predefined Mediterranean diet adherence with CVD in a UK general population setting. In this UK cohort, overall, we observed an inverse association of adherence to the Mediterranean diet with incident CVD and all-cause mortality. Our findings suggest that the MDS based on the Mediterranean dietary pyramid had the strongest associations with cardiovascular outcomes. Moreover, we report that other scoring algorithms of the Mediterranean diet that are based on dietary cut-offs in prior literature and on cohort tertiles may also be useful in the UK population. We further estimated that 3.9 % of total CVD incidence, 8.5 % of IHD or stroke incidence, and 12.5 % of CVD mortality in the EPIC-Norfolk cohort could have been avoided by increasing adherence to the Mediterranean diet. The findings indicate that adherence to the Mediterranean diet may contribute to a strategy for the primary prevention of CVD in the UK.

### Comparison with other studies

The modest degree of inverse association between adherence to the Mediterranean diet and incidence of CVD, CVD mortality, and all-cause mortality observed in EPIC-Norfolk is broadly in line with other published studies on the Mediterranean diet and CVD [11, 14, 38–44]. Specifically in the UK context, the Whitehall II study (n = 7731) concluded potential cardio-metabolic benefits of a Mediterranean-like diet for British adults, but did not find any significant associations after adjustment for confounders and did not assess the Mediterranean diet per se, by inferring the diet based on data-driven cluster analysis [45]. A few other studies in non-Mediterranean countries examined the association of pre-defined Mediterranean diet with CVD [12–14, 41–43, 46]. While their findings were broadly consistent with our findings, estimates of associations were often imprecise with wide confidence intervals or were non-significant. Exceptionally, one recent US study based on a large multi-ethnic cohort (n = 215,782) found that mMDS was associated with 11 to 28 % lower CVD mortality [43]. Based on our findings evaluating different MDSs, the inverse association could be stronger, if the study evaluated the other MDSs better suited to a non-Mediterranean population than mMDS. However, compared to studies conducted in Mediterranean cohorts [40, 47, 48], our estimates appear modest, which could be reflective of the fact that high adherence to a Mediterranean diet in this UK cohort might still not be fully representative of a traditional Mediterranean diet, as might be observed in the Mediterranean regions.

PAF was not estimated from any prior prospective studies, but can be manually calculated in the PREDIMED trial for incidence of either IHD or stroke [11]. The control group of this high-risk trial in Spain had an incidence of the composite outcome of 11.2 per 1000 person-years, similar to the incidence of our overall cohort (12.0 per 1000 person-years). According to effect estimates reported in the publication, the PAF for the outcome for no intervention in the PREDIMED trial was estimated to be 27.8 %, whereas PAF in the EPIC-Norfolk cohort for suboptimal adherence to PyrMDS was 8.5 %. The corresponding number of cases preventable and the number needed to treat over 10 years of follow-up were estimated to be 28.5 per 1000 population and 35.1, respectively, in PREDIMED and 9.2 per 1000 and 108.9, respectively, in EPIC-Norfolk.

One possible explanation for this discrepancy in these measures between PREDIMED and EPIC-Norfolk could be bias toward the null in the EPIC-Norfolk cohort, because of measurement errors and temporal changes in diet. Moreover, despite the inverse association with CVD incidence, the variability of adherence to the Mediterranean diet might not fully capture a high-quality diet in the UK. Indeed, none of the participants recorded the optimal score of PyrMDS (the observed highest = 13.1; the possible highest = 15.0). Alternatively, as mentioned above, the difference could reflect that the PREDIMED trial compared the Mediterranean diet against a non-Mediterranean diet, whereas EPIC-Norfolk tested a diet not fully in line with the Mediterranean diet.

### Interpretation of results and implications

Evidence from the Lyon Diet Heart Study in France and the more recent PREDIMED trial in Spain supports the causal effect of adherence to the Mediterranean diet on CVD outcomes [10, 11, 49, 50]. The PAF estimates in EPIC-Norfolk suggest that 1 to 6 % of all CVD, and 2 to 14 % of IHD or stroke incidence could be avoided by adhering to the Mediterranean diet in both a general population and a high-risk population, defined by QRISK2, for whom statin prescription would be recommended in the UK [36]. Of note, statin treatment could prevent 24 % of new-onset CVD, according to trials in the UK [51]. However, while statins do not influence other outcomes or may even increase risk of diabetes [52], greater adherence to the Mediterranean diet by contrast may have benefits beyond prevention of CVD. Our estimates are comparable to the corresponding PAF estimates for physical inactivity, which were 5.8 % for IHD mortality and 9.4 % for all-cause mortality [53].

While an alternative healthy dietary pattern beyond the Mediterranean diet may exist due to cultural differences, our PAF estimates indicate that the Mediterranean diet should be one option for a healthy diet in the UK, where CVD accounts for approximately 155,000 deaths, or more than a quarter of all deaths each year, and associated healthcare costs are estimated to be £11 billion per year and increasing [54]. If we assume causality and generalizability of our findings to the general UK population, a PAF of 12.5 % would have equated to 19,375 cases of CVD deaths preventable each year. Further investigation is warranted to explore the effectiveness of a population-level recommendation and cost-effectiveness of the Mediterranean diet in the UK and other non-Mediterranean countries, as a component of lifestyle recommendations for both the general and high CVD risk populations.

The use of FFQ as the dietary assessment instrument in our study limits the ability to precisely measure adherence to the Mediterranean diet, as it did not differentiate between extra virgin olive oil and other olive oils or between tree nuts (legumes) and peanuts, as evaluated in the PREDIMED trial [4]. Nonetheless, this study indicates utility of three scoring algorithms for the MDS: PyrMDS, LitMDS and tMDS, to represent adherence to a Mediterranean-style dietary pattern in epidemiological settings. Of note, findings are similar upon alternative categorisations of food groups for the MDSs, and upon other sensitivity analyses (Additional file 1: Table S7). Our findings indicate that using sex-specific medians (mMDS) in a given population would be too crude and not sensitive enough to assess adherence to the diet in a non-Mediterranean country. On the other hand, PyrMDS and LitMDS may deserve future investigation for clinical application, as they allowed calculation of MDS without use of reference population levels (e.g. medians or tertiles) [5]. PyrMDS is likely to be better than the others because the scoring accounts for the continuous property of dietary consumption, for the contemporary food environment distinguishing between processed and unprocessed meat, and for available evidence from epidemiological studies by encouraging moderate consumption of fermented dairy products, as originally characterised by the Mediterranean diet [2, 55, 56]; of note, dairy consumption was considered as an adverse component in mMDS and tMDS. Our findings guide further studies to investigate the utility of the Mediterranean dietary pyramid (and PyrMDS) for clinical practice and public health promotion in both Mediterranean and non-Mediterranean populations.

### Strengths and limitations

These results are of interest as the first to extensively examine the association of the Mediterranean diet with CVD in the UK and estimate the population impact of increasing adherence. Evaluation of four different MDSs clarified the difference between their characteristics and their utility in a non-Mediterranean context. A strength of this study is that it included a large sample size with long follow-up time. Because outcome ascertainment in this cohort was externally linked to medical records, it also minimised bias that could arise during follow-up. As limitations, measurement errors were present in our self-reported dietary and covariate measurements, and we could not fully account for changes in diet throughout the study period, although the errors were reduced by using repeated measures of diet, as well as time-varying covariates [27, 46]. We considered BMI as a confounder in our analyses, although recent evidence suggests potential mediating effects of BMI in the diet CVD association [57], and this could lead to underestimation of our effect estimates. However, this is unlikely given the consistency of our estimates across the levels of adjustment. Residual confounding is possible, because of unmeasured confounders and imprecise measurement of potential confounders. Generalisability is limited because of potential healthy cohort bias [23], dietary measures collected more than 15 years ago, and inclusion of largely white European individuals in the UK.

## Conclusions

We observed a lower incidence of CVD with higher adherence to the Mediterranean diet in this UK cohort. Our study also informs potential population impact of increasing adherence to the Mediterranean diet in a UK population. These results add to the pool of evidence on the health benefits of the Mediterranean diet, even in a non-Mediterranean country where an optimal dietary pattern is unknown. Our findings stimulate future population-based and clinical investigations into the efficacy and effectiveness of adhering to the Mediterranean diet in contemporary, non-Mediterranean populations.

## Additional file

Additional file 1:
**Text S1.** Scoring method of the four Mediterranean diet scores. **Table S1.** Mediterranean dietary pattern scores, components and corresponding food frequency questionnaire items used in EPIC-Norfolk. **Table S2.** Pyramid based Mediterranean diet score (PyrMDS) scoring criteria. **Table S3.** Characteristics of dietary consumption of components of the Mediterranean diet at baseline and follow-up among 23,902 adults in EPIC-Norfolk. **Table S4.** Prospective association between fifths of the degree of adherence to the Mediterranean diet and incident cardiovascular diseases in EPIC-Norfolk (n = 23,902, 7606 cases/269,935 person-years). **Table S5.** Associations of adherence to the Mediterranean diet with incident CVD when two measures of the adherence were evaluated simultaneously for comparison: EPIC-Norfolk Study. **Table S6.** Cardiovascular disease incidence or mortality and all-cause mortality, the number of cases and proportion preventable by increasing adherence to the Mediterranean diet to the top third of the Mediterranean dietary score based on the dietary pyramid: the EPIC-Norfolk cohort. **Table S7.** Prospective association between adherence to the Mediterranean diet and incident cardiovascular diseases in EPIC-Norfolk: sensitivity analysis to examine robustness of the findings across different analytical approaches. **Figure S1.** Prospective association between adherence to the Mediterranean diet and incidence of cardiovascular diseases in EPIC-Norfolk: sensitivity analysis to examine influence of each component of the Mediterranean diet. (DOCX 215 kb)

## Abbreviations

BMI: Body mass index
CI: Confidence interval
CVD: Cardiovascular diseases
EPIC: European Prospective Investigation of Cancer
FFQ: Food frequency questionnaire
HR: Hazard ratio
IHD: Ischaemic heart disease
LitMDS: Literature based MDS
MDS: Mediterranean diet score
PyrMDS: Pyramid based MDS
mMDS: median based MDS
PAF: Population attributable fraction
PREDIMED: Prevención con Dieta Mediterránea
SD: Standard deviation
tMDS: tertile based MDS
UK: United Kingdom
