# Supplementary material for: Prospective association of the Mediterranean diet with cardiovascular disease incidence and mortality and its population impact in a non-Mediterranean population: the EPIC-Norfolk study
Source: BMC Med. 2016 Sep 29;14:135. doi: 10.1186/s12916-016-0677-4 (PMC5041408; doi:10.1186/s12916-016-0677-4)
Supplement: Additional file 1: — Text S1. Scoring method of the four Mediterranean diet scores. Table S1. Mediterranean dietary pattern scores, components and corresponding food frequency questionnaire items used in EPIC-Norfolk. Table S2. Pyramid based Mediterranean diet score (PyrMDS) scoring criteria. Table S3. Characteristics of dietary consumption of components of the Mediterranean diet at baseline and follow-up among 23,902 adults in EPIC-Norfolk. Table S4. Prospective association between fifths of the degree of adherence to the Mediterranean diet and incident cardiovascular diseases in EPIC-Norfolk (n = 23,902, 7606 cases/269,935 person-years). Table S5. Associations of adherence to the Mediterranean diet with incident CVD when two measures of the adherence were evaluated simultaneously for comparison: EPIC-Norfolk Study. Table S6. Cardiovascular disease incidence or mortality and all-cause mortality, the number of cases and proportion preventable by increasing adherence to the Mediterranean diet to the top third of the Mediterranean dietary score based on the dietary pyramid: the EPIC-Norfolk cohort. Table S7. Prospective association between adherence to the Mediterranean diet and incident cardiovascular diseases in EPIC-Norfolk: sensitivity analysis to examine robustness of the findings across different analytical approaches. Figure S1. Prospective association between adherence to the Mediterranean diet and incidence of cardiovascular diseases in EPIC-Norfolk: sensitivity analysis to examine influence of each component of the Mediterranean diet. (DOCX 215 kb) [file 12916_2016_677_MOESM1_ESM.docx]

Additional file 1

[eText 1: Scoring method of the four Mediterranean diet scores 3](#_Toc450227741)

[eTable 1: Mediterranean dietary pattern scores, components and corresponding food frequency questionnaire items used in EPIC-Norfolk. 4](#_Toc450227742)

[eTable 2: Pyramid based Mediterranean diet score (PyrMDS) scoring criteria. 5](#_Toc450227743)

[eTable 3: Characteristics of dietary consumption of components of the Mediterranean diet at baseline and follow-up among 23,902 adults in EPIC-Norfolk. 6](#_Toc450227744)

[eTable 4: Prospective association between fifths of the degree of adherence to the Mediterranean diet and incident cardiovascular diseases in EPIC-Norfolk (n=23,902, 7,606cases / 269,935 person-years). 7](#_Toc450227745)

[eTable 5: Associations of adherence to the Mediterranean diet with incident CVD when two measures of the adherence were evaluated simultaneously for comparison: EPIC-Norfolk Study^1^. 8](#_Toc450227746)

[eTable 6: Cardiovascular disease incidence or mortality and all-cause mortality, the number of cases and proportion preventable by increasing adherence to the Mediterranean diet to the top third of the Mediterranean dietary score based on the dietary pyramid: the EPIC-Norfolk cohort^1^ 9](#_Toc450227747)

[eTable 7: Prospective association between adherence to the Mediterranean diet and incident cardiovascular diseases in EPIC-Norfolk: sensitivity analysis to examine robustness of the findings across different analytic approach. 10](#_Toc450227748)

[eFigure 1: Prospective association between adherence to the Mediterranean diet and incidence of cardiovascular diseases in EPIC-Norfolk: 11](#_Toc450227749)

[References 12](#_Toc450227750)

# Text S1: Scoring method of the four Mediterranean diet scores

For PyrMDS, fifteen components were derived based on the pyramid, for which continuous scores of 0 to 1 were assigned for each component according to the participant’s degree of adherence to the recommendation (eTable 2). For components for which high consumption was recommended (vegetables, legumes, fish), 0 to 1 points were assigned proportionally from no consumption to the recommended level of consumption. This scoring was reversed for components for which low consumption was recommended (red meat, processed meat, potato, sweets). For components for which moderate consumption was recommended (fruits, cereals, nuts, eggs, dairy, white meat), we assigned a score of 1 for consumption within the recommendation levels and 0 for no consumption. Overconsumption two-fold higher than the mid-point of the recommended intake was penalised and received a maximum of 0.5 point. For alcohol, sex-specific cut-offs were determined and moderate consumption was given 1 point; no consumption, 0.5 point; and overconsumption, 0 point [1].

Sofi *et al*. developed a MDS based on literature (LitMDS), after systematically reviewing published cohort studies [2]. The authors estimated weighted median intakes of nine Mediterranean diet components (vegetables, legumes, fruit and nuts, cereals, dairy, fish, meat, alcohol and olive oil) in different cohorts, and determined absolute cut-off points (with the exception of olive oil) to assign scores of 0, 1 or 2 for each component [2]. For olive oil, the authors suggested 0 point for occasional use, 1 for frequent use and 2 for regular use [2], and we assigned 0 to all non-consumers, 1 for below median and 2 for above median levels of olive oil intake.

The third MDS (mMDS) was derived from relative cut-offs (sex-specific medians) of consumption of nine dietary components [3,4] with a possible range from 0 to 9 points. For each of six components (vegetables, legumes, fruit and nuts, cereals, fish, ratio of monounsaturated and polyunsaturated fatty acids to saturated fatty acids), 1 point was assigned for consumption above the sex-specific median; and 0, otherwise. Scoring was reversed for total dairy products and meat products, so that 1 point was assigned for consumption below the median; but 0 point, otherwise. For alcohol consumption, 1 point was assigned for moderate ethanol consumption (men, 10 to 50 g/day; women, 5-25 g/day); and 0 otherwise. The fourth MDS was derived from sex-specific tertiles (tMDS) (range 0-18) based on loosely the same nine dietary components as above, and assigned scores of 0, 1 or 2 for each component [5], with minor modifications. For alcohol, for instance, 2 points were assigned for moderate consumption (men, 10 to 50 g/day of ethanol; women, 5 to 25 g/day) and 0 point was assigned for non-consumers and high consumers. For olive oil intake, which had highly skewed distribution, 0 point was given for no consumption, 1 for consumption below the median among consumers, and 2 for consumption above the median.

# Table S1: Mediterranean dietary pattern scores, components and corresponding food frequency questionnaire items used in EPIC-Norfolk.

| **Food groups** | **Items listed in the Food Frequency Questionnaire** | **Mediterranean diet score (score range)^1^** | | | |
| --- | --- | --- | --- | --- | --- |
|  |  | **PyrMDS, based on the Mediterranean diet pyramid (0-15)** | **LitMDS, based on published literature (0-18)** | **mMDS, based on medians of dietary intakes (0-9)** | **tMDS, based on tertiles of dietary intakes (0-18)** |
| Vegetables | Carrots, spinach, broccoli/spring greens/kale, brussels sprouts, cabbage, marrow/courgettes, cauliflower, parsnips/turnips/swedes, leeks, onions, garlic, mushrooms, sweet peppers, green salad/lettuce/cucumber/celery, watercress, tomatoes, sweetcorn, beetroot, coleslaw, avocado, vegetable soups, ketchup, pickles | 0 to 1 | 0, 1, or 2 | 0 or 1 | 0, 1, or 2 |
| Legumes | Peas, green beans/broad beans/runner beans, beansprouts, baked beans, dried lentils/beans/peas, tofu/soya meat/TVP/vegeburger | 0 to 1 | 0, 1, or 2 | 0 or 1 | 0, 1, or 2 |
| Fruits*^2^* | Apples, pears, oranges/satsumas/mandarins, grapefruits, bananas, grapes, melon, peaches/plums/apricots, strawberries/raspberries/kiwi, tinned fruits, dried fruits, | 0 to 1  0 to 1 | 0, 1, or 2 | 0 or 1 | 0, 1, or 2 |
| Nuts*^2^* | Peanuts and other nuts, peanut butter |  |  |  |  |
| Cereals | Brown bread and rolls, wholemeal bread and rolls, crispbread, porridge/readybrek, breakfast cereal, brown rice, wholemeal pasta, white bread and rolls, cream crackers/cheese biscuits, white rice, white or green pasta, lasagne/moussaka, pizza | 0 to 1 | 0, 1, or 2 | 0 or 1 | 0, 1, or 2 |
| Dairy | Single or sour cream, double or clotted cream, low fat yoghurt/fromage frais, full fat or Greek yoghurt, dairy desserts, cheese, cottage cheese, milk | 0 to 1 | 0, 1, or 2 | 0 or 1 | 0, 1, or 2 |
| Fish | Fried fish, fish fingers/fish cakes, other white fish, oily fish, shellfish, fish roe/taramasalata | 0 to 1 | 0, 1, or 2 | 0 or 1 | 0, 1, or 2 |
| Red meats*^2^* | Beef, pork, lamb, beefburgers, meat soups (if red meats e.g. oxtail soup) | 0 to 1  0 to 1  0 to 1 | 0, 1, or 2 | 0 or 1 |  |
| Processed meats*^2^* | Bacon, ham, corned beef, sausages, savoury pie, liver, liver pate and liver sausage |  |  |  | 0, 1, or 2 |
| White meats*^2^* | Chicken and other poultry, meat soups (if white meats e.g. chicken soup) |  |  |  |  |
| Eggs | Eggs, quiche | 0 to 1 |  |  |  |
| Potatoes | Boiled/mashed/instant/jacket potatoes, chips, roast potatoes, potato salad | 0 to 1 |  |  |  |
| Alcohol/Ethanol*^3^* | Wine, beer/lager/cider, port/sherry/vermouth/liqueurs, spirits | 0 to 1 | 0, 1, or 2 | 0 or 1 | 0, 1, or 2 |
| Sweets | Sweet biscuits (chocolate), sweet biscuits (plain), cakes (homemade/ready made), buns/pastries cakes (homemade/ready made), fruits pies etc (homemade/ready made), sponge puddings (homemade/ready made), milk puddings, ice cream, chocolate, chocolate snacks, sweets/toffees/mints, sugar added to tea/coffee/cereal, jam/marmalade/honey, low cal/diet fizzy drinks, fizzy soft drinks, fruit squash/cordial | 0 to 1 |  |  |  |
| Fat*^4^* | Olive oil, monounsaturated fatty acids, polyunsaturated fatty acids, saturated fatty acids | 0 to 1 | 0, 1, or 2 | 0 or 1 | 0, 1, or 2 |

*^1^* See methods and eTable 2 for further details on scoring criteria for the four Mediterranean diet scores.

*^2^* In LitMDS, mMDS and tMDS, fruits and nuts were aggregated as a single component. All meat components were aggregated as well for these three scores. *^3^* Included in in PyrMDS as alcohol (beverage), and in LitMDS, mMDS and tMDS as ethanol (nutrient).
*^4^* Included in PyrMDS, LitMDS and tMDS as olive oil, and in mMDS as ratio of the sum of monounsaturated fatty acids and polyunsaturated fatty acids to saturated fatty acids.

# Table S2: Pyramid based Mediterranean diet score (PyrMDS) scoring criteria.

| **Component** | **Recommended intake***^1^* | **Score of 0***^1^* | **Score of 1***^1^* |
| --- | --- | --- | --- |
| Vegetables*^2^* | ≥6/d | 0/d | ≥6/d |
| Legumes*^2^* | ≥2/wk | 0/wk | ≥2/wk |
| Fruits*^3^* | 3-6/d | 0/d | 3-6/d |
| Nuts*^3^* | 1-2/d | 0/d | 1-2/d |
| Cereals*^3^* | 3-6/d | 0/d | 3-6/d |
| Dairy*^3^* | 2/d | 0/d | 1.5-2.5/d |
| Fish*^2^* | ≥2/wk | 0/wk | ≥2/wk |
| Red meat*^5^* | ˂2/wk | ≥4/wk | ˂2/wk |
| Processed meat*^5^* | ≤1/wk | ≥2/wk | ≤1/wk |
| White meat*^3^* | 2/wk | 0/wk | 1.5-2.5/wk |
| Egg*^3^* | 2-4/wk | 0/wk | 2-4/wk |
| Potato*^5^* | ≤3/wk | ≥6/wk | ≤3/wk |
| Sweets*^5^* | ≤2/wk | ≥4/wk | ≤2/wk |
| Alcohol*^4^* | 2/d for men,  1/d for women | ≥4/d for men,  ≥2/d for women | 1.5-2.5/d for men,  0.5-1.5/d for women |
| Olive oil*^6^* | Principal source of  dietary lipids | Non-consumers | Consumers |

*^1^* All recommendations are in number of servings per day or per week and we used continuous scoring for all components except olive oil.
*^2^* For these components for which a high consumption was recommended, continuous scores from 0 to 1 were assigned proportionally from no consumption to meeting the recommended level of consumption.
*^3^* For components for which moderate consumption was recommended, we assigned a score of 1 for consumption within the recommendation levels and 0 for no consumption, with consumption levels in between scored proportionately. Overconsumption (double the mid-point value of the recommended intake) was penalised and received a maximum score of 0.5, with consumption between the recommended level and the penalty point scored proportionally.
*^4^* For alcohol we assigned a score of 1 for consumption levels within recommendation. Non-consumption was scored 0.5 while overconsumption was scored 0.
*^5^* For these components for which a low consumption was recommended, consumption below the recommended levels was assigned a score of 1 and double the recommended levels were assigned a score of 0, with levels in between scored proportionally.
*^6^* For olive oil all non-consumers were scored 0 and all consumers 1.

Table S3: Characteristics of dietary consumption of components of the Mediterranean diet at baseline and follow-up among 23,902 adults in EPIC-Norfolk.

| **Dietary variables***^1^* | **Median [IQR]**  **at baseline**  **n=23,694** | **Median [IQR] during follow-up n=11,466** | **Mean change (SD) between two FFQs***^2^* |  | **Correlation coefficient (Spearman's rho)** | | | | |
| --- | --- | --- | --- | --- | --- | --- | --- | --- | --- |
|  |  |  |  |  | **Between two repeated measures** | **with Mediterranean diet score at baseline** | | | |
|  |  |  |  |  |  | **PyrMDS** | **LitMDS** | **mMDS** | **tMDS** |
| **Foods and nutrients** |  |  |  |  |  |  |  |  |  |
| Vegetables | 223 [158, 301] | 230 [165, 312] | 3.6 (106) |  | 0.64 | 0.37 | 0.43 | 0.49 | 0.51 |
| Legumes | 54 [34, 78] | 51 [32, 76] | -1.8 (38) |  | 0.47 | 0.13 | 0.21 | 0.37 | 0.39 |
| Fruit and nuts | 213 [127, 327] | 240 [149, 360] | 17.1 (165) |  | 0.65 | 0.45 | 0.53 | 0.43 | 0.45 |
| Fruits | 210 [125, 323] | 236 [145, 356] | 17.0 (164) |  | 0.64 | 0.44 | 0.53 | 0.42 | 0.45 |
| Nuts | 0 [0, 3] | 0 [0, 3] | 0.2 (7.5) |  | 0.59 | 0.3 | 0.18 | 0.16 | 0.2 |
| All grains | 156 [112, 211] | 152 [109, 205] | -7.2 (79) |  | 0.51 | 0.32 | 0.48 | 0.41 | 0.4 |
| All meat | 98 [69, 131] | 94 [65, 128] | -0.1 (48) |  | 0.56 | -0.29 | -0.36 | -0.26 | -0.26 |
| White meat | 22 [14, 49] | 23 [14, 49] | 0.8 (25) |  | 0.51 | 0.18 | -0.02 | 0.02 | 0.02 |
| Red meat | 37 [21, 59] | 33 [18, 55] | -1.3 (31) |  | 0.59 | -0.4 | -0.33 | -0.28 | -0.26 |
| Processed meat | 25 [14, 38] | 24 [13, 38] | 1.5 (21) |  | 0.57 | -0.31 | -0.3 | -0.23 | -0.23 |
| Fish | 33 [21, 48] | 33 [22, 49] | 1.3 (26) |  | 0.51 | 0.35 | 0.39 | 0.38 | 0.4 |
| Dairy | 399 [296, 523] | 400 [294, 529] | 1.3 (157) |  | 0.60 | 0.03 | -0.09 | -0.27 | -0.24 |
| Egg | 13 [6, 23] | 14 [6, 24] | 0.9 (16) |  | 0.48 | 0.25 | 0.04 | 0.03 | 0.05 |
| Potato | 113 [82, 144] | 112 [82, 144] | 0.5 (63) |  | 0.44 | -0.32 | -0.1 | -0.1 | -0.08 |
| Sweet | 166 [110, 238] | 165 [108, 236] | 4.6 (174) |  | 0.56 | -0.12 | -0.14 | -0.17 | -0.17 |
| Ethanol | 4.1 [0.8, 10.9] | 4.8 [0.9, 11.7] | 0.5 (7.8) |  | 0.85 | 0.14 | 0.04 | 0.23 | 0.3 |
| Olive oil*^3^* | 1 [0.6, 1.5] | 1 [0.6, 1.5] | 0.02 (1.3) |  | 0.53 | 0.39 | 0.42 | 0.06 | 0.33 |
| Monounsaturated fats | 25 [22, 28] | 25 [21, 28] | 0.06 (5.1) |  | 0.50 | -0.21 | -0.34 | -0.3 | -0.32 |
| Polyunsaturated fats | 13 [10, 16] | 13 [11, 16] | 0.05 (4.7) |  | 0.49 | 0.16 | 0.13 | 0.28 | 0.13 |
| Saturated fats | 27 [23, 32] | 27 [23, 31] | -0.4 (6.3) |  | 0.60 | -0.29 | -0.4 | -0.52 | -0.41 |
| Total energy | 1985 [1629, 2403] | 1900 [1570, 2298] | -92.5 (510) |  | 0.62 | -0.3 | -0.33 | -0.01 | -0.24 |
| **Mediterranean diet score (score range)** *^4^* |  |  |  |  |  |  |  |  |  |
| PyrMDS, based on dietary pyramid (0-15) | 8.4 [7.5, 9.3] | 8.7 [7.8, 9.5] | 0.12 (1.1) |  | 0.6 | 1 | 0.68 | 0.53 | 0.64 |
| LitMDS, based on literature (0-18) | 10 [8, 11] | 10 [9, 12] | 0.43 (2.2) |  | 0.56 | 0.68 | 1 | 0.66 | 0.8 |
| mMDS, based on medians (0-9) | 4 [3, 6] | 4 [3, 6] | 0.05 (1.7) |  | 0.52 | 0.53 | 0.66 | 1 | 0.81 |
| tMDS, based on tertiles (0-18) | 8 [6, 10] | 9 [7, 11] | 0.42 (2.6) |  | 0.57 | 0.64 | 0.8 | 0.81 | 1 |

Abbreviations: FFQ, food-frequency questionnaire; IQR, interquartile range.

*^1^* The list includes dietary variables that were used in one or more of the Mediterranean diet scores. All intakes are in gram/day (apart from total energy, kcal/day) and adjusted to a 2,000 kcal/day diet. Baseline values were used for assessment of correlation with Mediterranean diet score at baseline.

*^2^* Assessed based on 11,258 participants (4,717 men and 6,541 women) who had both FFQ1 and FFQ2 data. The median duration between 2 FFQ was 3.7 years [IQR 3.1, 4.0].

*^3^* Intake based on 1,853 olive oil consumers (867 men and 987 women) only for FFQ1 and 1,653 consumers (692 men and 961 women) only for FFQ2 due to large numbers of non-consumers of olive oil in this cohort.

*^4^* Ordinal scores were assigned to participants, according to four different pre-specified algorithms (see methods, eTable 1 and eTable 2 for details).

# Table S4: Prospective association between fifths of the degree of adherence to the Mediterranean diet and incident cardiovascular diseases in EPIC-Norfolk (n=23,902, 7,606cases / 269,935 person-years).

| **Mediterranean diet score***^1^* | **Hazard ratios (95% confidence intervals)** | | |
| --- | --- | --- | --- |
|  | **Adjusted for age and sex** | **Further adjusted for potential confounders***^2^* | **Further adjusted for potential mediators***^2^* |
| **Scoring based on the Mediterranean-diet pyramid (PyrMDS: 0-15)***^3^* |  |  |  |
| Q1 (3.2-7.4) | Reference | Reference | Reference |
| Q2 (7.4-8.2) | 0.95 (0.89, 1.02) | 0.95 (0.89, 1.02) | 0.95 (0.89, 1.01) |
| Q3 (8.2-8.9) | 0.94 (0.87, 1.00) | 0.95 (0.88, 1.01) | 0.94 (0.88, 1.01) |
| Q4 (8.9-9.6) | 0.89 (0.83, 0.95) | 0.90 (0.83, 0.96) | 0.90 (0.84, 0.97) |
| Q5 (9.6-13.1) | 0.80 (0.74, 0.86) | 0.85 (0.79, 0.92) | 0.86 (0.80, 0.93) |
| *p*-trend | <0.001 | <0.001 | <0.001 |
|  |  |  |  |
| **Scoring based on published literature (LitMDS: 0-18)***^3^* |  |  |  |
| Q1 (0-5) | Reference | Reference | Reference |
| Q2 (6-7) | 0.96 (0.90, 1.03) | 0.98 (0.92, 1.04) | 0.97 (0.91, 1.03) |
| Q3 (8-9) | 0.91 (0.84, 0.98) | 0.91 (0.84, 0.98) | 0.91 (0.84, 0.98) |
| Q4 (10-11) | 0.93 (0.86, 1.00) | 0.92 (0.85, 1.00) | 0.92 (0.85, 1.00) |
| Q5 (12-18) | 0.87 (0.81, 0.94) | 0.90 (0.84, 0.97) | 0.90 (0.83, 0.97) |
| *p*-trend | <0.001 | 0.002 | 0.002 |
|  |  |  |  |
| **Scoring based on medians of dietary intakes (mMDS: 0-9)***^3^* |  |  |  |
| Q1 (0-2) | Reference | Reference | Reference |
| Q2 (3) | 0.97 (0.90, 1.05) | 0.97 (0.90, 1.05) | 0.97 (0.90, 1.05) |
| Q3 (4) | 0.92 (0.86, 0.99) | 0.94 (0.87, 1.01) | 0.93 (0.86, 1.00) |
| Q4 (5) | 0.93 (0.86, 1.00) | 0.95 (0.88, 1.02) | 0.94 (0.88, 1.02) |
| Q5 (6-9) | 0.93 (0.87, 1.00) | 0.96 (0.89, 1.03) | 0.95 (0.88, 1.02) |
| *p*-trend | 0.04 | 0.24 | 0.17 |
|  |  |  |  |
| **Scoring based on tertiles of dietary intakes (tMDS: 0-18)***^3^* |  |  |  |
| Q1 (0-5) | Reference | Reference | Reference |
| Q2 (6-7) | 0.92 (0.86, 0.99) | 0.92 (0.86, 0.99) | 0.93 (0.87, 1.00) |
| Q3 (8-9) | 0.93 (0.86, 0.99) | 0.94 (0.87, 1.01) | 0.93 (0.87, 1.00) |
| Q4 (10-11) | 0.88 (0.82, 0.95) | 0.88 (0.82, 0.95) | 0.88 (0.82, 0.95) |
| Q5 (12-18) | 0.90 (0.83, 0.97) | 0.92 (0.84, 1.00) | 0.91 (0.84, 0.99) |
| *p*-trend | 0.003 | 0.02 | 0.008 |

*^1^* For each of the Mediterranean diet scores, five groups (Q1 to Q5) were assigned to ensure approximately equal numbers of observations per group.

*^2^* See methods for list of confounders and mediators.
*^3^* Ordinal scores were assigned to participants, according to four different pre-specified algorithms (see methods, eTable 1 and eTable 2 for details).

# Table S5: Associations of adherence to the Mediterranean diet with incident CVD when two measures of the adherence were evaluated simultaneously for comparison: EPIC-Norfolk Study^1^.

| **MDS and basis for scoring (possible range)** | **Hazard ratio (95% confidence interval)***^1^* | | | |
| --- | --- | --- | --- | --- |
|  | **adjusted for PyrMDS***^2^* | **adjusted for LitMDS***^2^* | **adjusted for mMDS***^2^* | **adjusted for tMDS***^2^* |
| PyrMDS, based on the Mediterranean diet pyramid (0-15)*^2^* | --- | 0.95 (0.92-0.98) | 0.94 (0.91-0.97)*^3^* | 0.95 (0.92-0.98) |
| LitMDS, based on published literature (0-18)*^2^* | 0.99 (0.96-1.03) | --- | 0.95 (0.92-0.98)*^3^* | 0.97 (0.93-1.01) |
| mMDS, based on medians of dietary intakes (0-9)*^2^* | 1.02 (0.99-1.04)*^3^* | 1.02 (0.99-1.05)*^3^* | --- | 1.03 (0.99-1.08)*^3^* |
| tMDS, based on tertiles of dietary intakes (0-18)*^2^* | 1.00 (0.97-1.03) | 0.99 (0.95-1.03) | 0.94 (0.90-0.98)*^3^* | --- |

*^1^* Values are hazard ratios (95% confidence intervals) per 1 standard deviation of each MDS.

*^2^* Ordinal scores were assigned to participants, according to four different pre-specified algorithms (see methods, eTable 1 and eTable 2 for details).

^3^ Two MDS at the row and column were significantly different (*p*<0.05).

# Table S6: Cardiovascular disease incidence or mortality and all-cause mortality, the number of cases and proportion preventable by increasing adherence to the Mediterranean diet to the top third of the Mediterranean dietary score based on the dietary pyramid: the EPIC-Norfolk cohort^1^

|  | **EPIC-Norfolk cohort**  **N=23902** | | |  | **EPIC-Norfolk high risk population***^2^* **N=15767** | | |
| --- | --- | --- | --- | --- | --- | --- | --- |
|  | **Incidence***^3^* | **Cases preventable***^3^* | **PAF% (95% CI)** |  | **Incidence***^3^* | **Cases preventable***^3^* | **PAF% (95% CI)** |
| **Incident CVD events** |  |  |  |  |  |  |  |
| All incident CVD | 248.6 | 5.6 | 2.2 (0.3, 4.2) |  | 334.4 | 7.4 | 2.2 (0.0, 4.4) |
| Incident IHD | 98.2 | 5.7 | 5.8 (0.6, 11.1) |  | 138.3 | 7.4 | 5.4 (0.0, 10.8) |
| Incident Stroke | 33.8 | 2.2 | 6.5 (-3.2, 16.2) |  | 48.1 | 2.9 | 6.0 (-4.1, 16.2) |
| Incident IHD or stroke | 120.3 | 7.8 | 6.5 (2.6, 10.5) |  | 168.9 | 10.0 | 5.9 (1.9, 10.0) |
| **Mortality events** |  |  |  |  |  |  |  |
| All-cause mortality | 138.4 | 5.2 | 3.8 (0.8, 6.8) |  | 191.3 | 7.6 | 4.0 (0.8, 7.1) |
| CVD mortality | 43.9 | 4.1 | 9.3 (2.9, 15.7) |  | 65.0 | 5.6 | 8.6 (1.8, 15.4) |
| IHD mortality | 21.1 | 2.7 | 12.9 (2.7, 23.1) |  | 31.3 | 4.0 | 12.7 (1.8, 23.5) |
| Stroke mortality | 13.2 | 0.4 | 2.8 (-9.5, 15.2) |  | 19.6 | 0.4 | 2.1 (-11.8, 16.0) |
| IHD or stroke mortality | 34.1 | 3.2 | 9.4 (1.5, 17.3) |  | 50.3 | 4.4 | 8.7 (0.5, 17.0) |

*^1^* Supplementary table 2 for further details on the scoring criteria for PyrMDS. The top third was 9.1 out of possible 15 points.
*^2^* High risk was defined as a QRISK2 score of 10% or above for 10 year risk of CVD, for whom a pharmacological intervention (statin treatment) is guided in the United Kingdom.
*^3^* Per 1000 population over 10 years. PAF, population attributable fraction, indicating proportion of cases attributable to the exposure of interest (low adherence to the Mediterranean diet).

# Table S7: Prospective association between adherence to the Mediterranean diet and incident cardiovascular diseases in EPIC-Norfolk: sensitivity analysis to examine robustness of the findings across different analytic approach.

| **Sensitivity analysis***^1^* | **N Cases /**  **person-years** | **Hazard ratio (95% confidence interval) for each Mediterranean diet score^2^** | | | |
| --- | --- | --- | --- | --- | --- |
|  |  | **PyrMDS, based on the Mediterranean diet pyramid**  **(0-15)***^3^* | **LitMDS, based on published literature**  **(0-18)***^3^* | **mMDS, based on medians of dietary intakes (0-18)***^3^* | **tMDS, based on tertiles of dietary intakes (0-18)***^3^* |
| **Dietary measurements** |  |  |  |  |  |
| Using baseline FFQ only*^4^* | 7,553 /  268,684 | 0.95 (0.92, 0.97) | 0.96 (0.94, 0.99) | 0.99 (0.96, 1.01) | 0.97 (0.95, 0.99) |
| Excluding adults with a  single FFQ only*^5^* | 3,123 /  91,207 | 0.96 (0.92, 0.99) | 0.96 (0.93, 1.00) | 0.98 (0.95, 1.02) | 0.96 (0.93, 1.00) |
| Excluding top and bottom 1st  percentile energy intake*^6^* | 7,427 /  264,167 | 0.94 (0.92, 0.97) | 0.96 (0.94, 0.98) | 0.99 (0.96, 1.01) | 0.97 (0.94, 0.99) |
| Excluding top and bottom 5th  percentile energy intake*^7^* | 6,772 /  241,585 | 0.94 (0.91, 0.96) | 0.95 (0.93, 0.98) | 0.98 (0.95, 1.00) | 0.96 (0.93, 0.98) |
|  |  |  |  |  |  |
| **Accounting for competing risk of non-cardiovascular mortality** | 7,626 /  341,619 | 0.96 (0.94, 0.98) | 0.97 (0.94, 0.99) | 0.99 (0.96, 1.01) | 0.97 (0.95, 1.00) |
|  |  |  |  |  |  |
| **Alternative grouping of food items in components***^8^* | 7,606 /  269,935 | 0.95 (0.92, 0.97) | 0.98 (0.95, 1.00) | 0.99 (0.97, 1.02) | 0.97 (0.94, 0.99) |
|  |  |  |  |  |  |
| **Additional adjustment for QRISK2^9^** | 7,606 /  269,935 | 0.95 (0.93, 0.97) | 0.96 (0.94, 0.99) | 0.99 (0.96, 1.01) | 0.97 (0.94, 0.99) |

*^1^* Sensitivity analyses based on one imputed dataset only, apart from dietary assessment method and additional adjustment of QRISK2 which was based on ten imputed datasets.

^2^ All hazard ratios were for per one standard deviation of each of the Mediterranean diet score (MDS), representing adherence to the Mediterranean diet, and adjusted for confounders (see methods).

*^3^* Ordinal scores were assigned to participants, according to four different pre-specified algorithms (see methods, eTable 1 and eTable 2 for details).

^4^ Baseline FFQ only refers to assessment of diet using only the baseline food frequency questionnaire (n=23,694), where person-years were calculated from baseline until incident CVD or censoring.

*^5^* Repeated measures only refers to assessment of diet using averages intakes of baseline and follow-up food frequency questionnaire of participants who completed both (n=11,258), where person-years were calculated from date of follow-up until incident CVD or censoring.

*^6^* Excluded 702 participants (n=23,455).

*^7^* Excluded 3,516 participants (n=21,733).

*^8^* We excluded non-fermented dairy products (milk, single or sour cream, double or clotted cream, dairy desserts), processed fish (fried fish, fingers/fish cakes), refined cereal products (white bread and rolls, cream crackers/cheese biscuits, white rice, white or green pasta, lasagne/moussaka, pizza) and non-wine alcohol (beer/lager/cider, port/sherry/vermouth/liqueurs, spirits).
*^9^* Adjusted for confounders, mediators and QRISK2 (see methods).

**
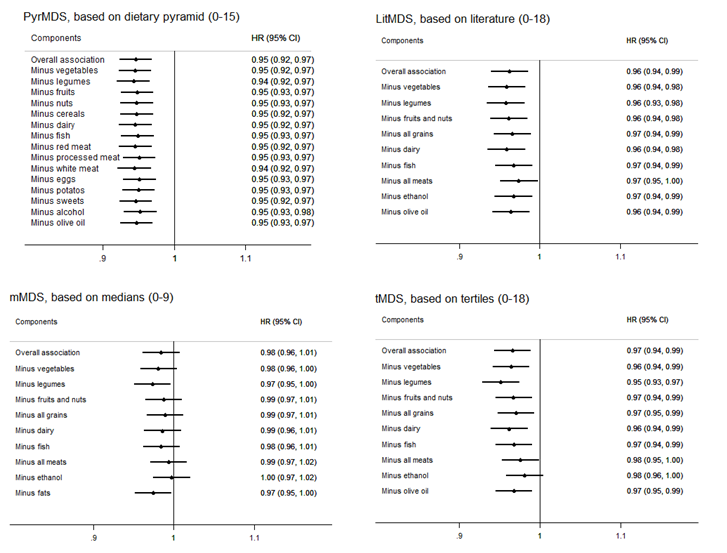
**
Figure S1: Prospective association between adherence to the Mediterranean diet and incidence of cardiovascular diseases in EPIC-Norfolk**:** sensitivity analysis to examine influence of each component of the Mediterranean diet. Adherence to the Mediterranean diet was scored by four different scores (see methods, eTable 1 and eTable 2 for details). Hazard ratio (HR) and 95% confidence interval (CI) were estimated per one standard deviation of each score (overall association) or of each score recreated after excluding one component of the score. All estimates shown are adjusted for confounders (see methods)

# References

1. Gronbaek M, Becker U, Johansen D, Gottschau A, Schnohr P, Hein HO, et al. Type of alcohol consumed and mortality from all causes, coronary heart disease, and cancer. Ann. Intern. Med. 2000;133:411–9.

2. Sofi F, Macchi C, Abbate R, Gensini GF, Casini A. Mediterranean diet and health status: an updated meta-analysis and a proposal for a literature-based adherence score. Public Health Nutr. 2014;17:2769–82.

3. Trichopoulou A, Orfanos P, Norat T, Bueno-de-Mesquita B, Ocké MC, Peeters PHM, et al. Modified Mediterranean diet and survival: EPIC-elderly prospective cohort study. BMJ. 2005/04/12 ed. 2005;330:991.

4. Trichopoulou A, Bamia C, Trichopoulos D. Mediterranean diet and survival among patients with coronary heart disease in Greece. Arch. Intern. Med. 2005;165:929–35.

5. The InterAct Consortium. Mediterranean Diet and Type 2 Diabetes Risk in the European Prospective Investigation Into Cancer and Nutrition (EPIC) Study: The InterAct project. Diabetes Care. 2011/07/27 ed. 2011;34:1913–8.
